# Functionalization of Lanthanated and Thoriated Tunsten Electrodes for pH Sensing

**DOI:** 10.1021/acsomega.5c06781

**Published:** 2025-09-17

**Authors:** Luis Díaz-Ballote, Elsy T. Vega-Lizama, Luis Maldonado, Román Castro

**Affiliations:** Department of Applied Physics, Center for Research and Advanced Studies, km 6 Carretera Antigua a Progreso, 97310 Cordemex, Mérida, Yucatán, México

## Abstract

The aim of this study was to evaluate the electrochemical performance of functionalized lanthanated (WLa) and thoriated (WTh) tungsten electrodes as pH sensors, with a focus on their sensitivity, hysteresis, and response time. The goal was to determine the effect of La_2_O_3_ or ThO_2_ combined with WO_3_ on the performance of the electrodes for general pH-sensing applications. A simple and low-cost functionalization method based on an oxidant flame from an oxyacetylene torch was used to produce a combined oxide film on the tungsten electrode. The electrochemical response of both electrodes was compared in terms of key parameters. The WLa electrode demonstrated higher sensitivity (−58.8 mV pH^–1^), a faster response time (18.4–19.4 s), and moderate hysteresis (12.1 mV), whereas the WTh electrode exhibited lower sensitivity (−41.6 mV pH^–1^), a response time of 19.8–21.1 s, and lower hysteresis (8.6 mV). X-ray photoelectron spectroscopy (XPS) analysis was conducted to explore the oxygen vacancies in both electrodes and their impact on electrode performance. The WLa electrode, with more oxygen vacancies, displayed superior sensitivity and a faster response time, making it well suited for general pH sensing. In contrast, the WTh electrode, with fewer oxygen vacancies, exhibited lower sensitivity but reduced hysteresis, which could be advantageous in applications where hysteresis is a critical factor. Both electrodes are durable and cost-effective alternatives to traditional sensing methods, offering high adaptability and reliability.

## Graphical abstract

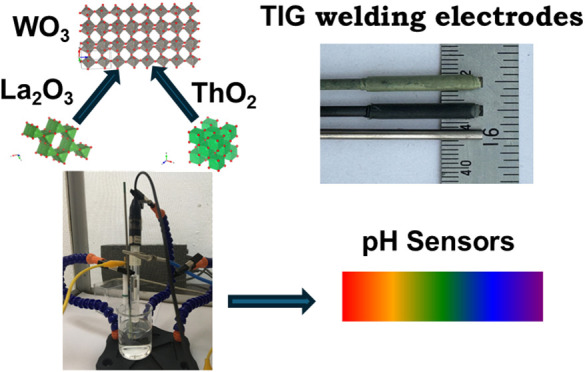

## Introduction

Low-cost and robust pH sensors are increasingly needed in healthcare, environmental monitoring, and industrial process control.^1,2^ While glass electrodes have long been the standard for pH measurement, their fragility and size restrict their use in dynamic or low-resource settings.^3,4^

Metal-oxide electrodes offer a compelling alternative owing to their mechanical durability, fabrication, and potential for miniaturization.^5^ Among these, tungsten trioxide (WO_3_) has demonstrated excellent sensitivity in acidic or alkaline environments.^6^ However, optimizing its nanostructure and chemical composition remains essential for achieving a near-Nernstian response, low hysteresis, and fast response times.^7^

In this context, we explore the use of rare earth oxide-doped WO_3_ coatings on tungsten inert gas (TIG) welding electrodes as a cost-effective sensing platform that leverages readily available industrial components.^8^ This may help reduce costs, potentially making metal-oxide-based pH electrodes more competitive with commercial glass electrodes. Additionally, these electrodes provide an accessible framework for investigating how rare-earth oxides (REOs) influence the pH-sensing behavior of WO_3_. In our previous studies,^9^ we examined pristine WO_3_ and WO_3_–CeO_2_ electrodes, finding that the incorporation of CeO_2_ led to encouraging improvements in the sensing response. These results motivated us to extend the investigation to other REOs, specifically La_2_O_3_ and ThO_2_, as potential modifiers of WO_3_ with the aim of enhancing its performance as a low-cost, robust pH sensor. Aligned with the principles of efficient material utilization and resource efficiency.

In this study, we evaluate the open-circuit potential (OCP) response of REO-doped WO_3_ electrodes across the pH range of 3–8, focusing on sensitivity, response time, and hysteresis. We demonstrate the feasibility of using REO-functionalized WO_3_ on TIG substrates as low-cost, high-performance pH sensors.

## Experimental Procedures

### Materials

Low-cost tungsten electrodes frequently used in tungsten inert gas (TIG) welding have been investigated for their potential application in pH sensing. The primary electrodes used consisted of tungsten with dispersed La_2_O_3_ or ThO_2_. These electrodes had a diameter of 3/32″ and a length of 7″ and contained between 1.8 and 2.2 wt % dispersed La_2_O_3_ or ThO_2_. The electrodes were acquired from “Yeswelder” through Amazon.com, with a cost of ∼3 USD (∼60 MXN) per 7-in. unit. Each electrode can be sectioned into seven or more smaller segments, allowing the fabrication of multiple sensors from a single electrode. Hereafter, the tungsten electrode codes are as follows: (1) WLa for tungsten coated with an oxide film of WO_3_ and La_2_O_3_ and (2) WTh for tungsten coated with an oxide film of WO_3_ and ThO_2_.

### Oxide Formation on WLa or WTh Electrodes

The tungsten electrodes, containing either dispersed La_2_O_3_ or ThO_2_, were functionalized by heating them via a widely accessible, well-known, and low-cost technique of working with metals: the oxy-acetylene welding/cutting technique. The process involved exposing electrodes to an oxidizing flame produced with a No. 0 nozzle tip. The flame temperature was estimated to reach 3000 °C.^10,11^ The flame was carefully swept across the electrode surface for a duration of 10 s, during which the electrode visibly glowed red. At this stage, the formation of a mixed oxide layer was achieved.^12^ Following the oxidation step, the oxidized electrode end was covered with heat shrink polytetrafluoroethylene (PTFE) except for 2 mm.^13^ This exposed section was then utilized as the pH sensor, while the opposite side of the rod was employed to connect the potential measuring device (a potentiostat).

### Preparation of pH Buffer Solutions

To prepare the pH buffer solutions, stock solutions were first created. A 0.1 M solution of citric acid monohydrate (C_6_H_8_O_7_·H_2_O) and a 0.2 M solution of dibasic sodium phosphate (Na_2_HPO_4_) were prepared.^14^ These stock solutions were then mixed at specific ratios to achieve a series of buffer solutions with distinct pH values. The final buffer solutions had pH values in the range of 2.5–8 and were used for subsequent sensor response testing. The pH of each solution was confirmed with a commercial pH meter (HI98103, HANNA Instruments).

### SEM and EDS Analysis of Tungsten Electrodes

The surface morphologies of the tungsten electrodes, specifically WLa and WTh, were examined via a JEOL JSM-7800F scanning electron microscope. The microscope was operated at an accelerating voltage of 15 kV, and images were captured in low-angle backscattered electron (LABE) mode. Additionally, energy-dispersive X-ray spectroscopy (EDS/EDAX) was conducted to obtain the elemental compositions of the electrode surfaces.

### X-ray Photoelectron Spectroscopy (XPS) Analysis

Surface characterization of the tungsten electrodes was conducted via X-ray photoelectron spectroscopy (XPS) with a Thermo Scientific K-Alpha spectrometer. The instrument was equipped with a monochromatic Al Kα radiation source operating at an energy of 1486.6 eV. The obtained XPS signals were analyzed and deconvoluted via a freeware program (XPSpeak version 4.1) developed by Raymond Kwok from Hong Kong, China.

### Open Circuit Potential Measurements

The open-circuit potential (OCP) response of the tungsten sensor as a function of pH was evaluated by measuring the potential between the sensor and an Ag/AgCl reference electrode. The measurements were conducted via a Gamry Potentiostat 600 series, with data acquisition and analysis performed via Gamry’s Echem Analyst software.

### Sensitivity

The sensitivity of the sensor was evaluated using buffer solutions with varying pH values. The sensor was immersed in each solution, and the OCP was recorded against an Ag/AgCl reference electrode. A platinum wire served as the auxiliary electrode to complete the conventional three-electrode electrochemical cell configuration; however, it did not contribute to the measurement itself. While the sensor remained immersed, the potential was continuously recorded over a period of 1200 s. After each measurement, the sensor was rinsed with deionized water, and the procedure was repeated three times in solutions with different pH values. This protocol was applied to both types of sensors: WLa and WTh.

### Hysteresis

To assess hysteresis, the same stock solutions were used to prepare buffers with different pH values. The evaluation began by simultaneously immersing the sensor and a commercial pH meter (Hanna) in a solution with a pH of 3.2. Under these conditions, the OCP was continuously monitored. Every 120 s, the pH of the solution was modified by the controlled addition of the stock solutions, with changes of approximately one pH unit. The pH sequence was 3.2 → 4.3 → 5.3 → 6.6, after which the direction was reversed, returning to the initial pH in the sequence of 6.6 → 5.3 → 4.3 → 3.2. The sensor remained in the solution throughout the entire procedure, and the measurements were uninterrupted. This evaluation was performed in triplicate at minimum.

### Response Time

The response time was evaluated using buffer solutions with pH values of 3.1, 4.9, and 7.3. Prior to each measurement, the sensor was rinsed with distilled water, gently blotted to remove excess moisture, and then immersed in the test solution. Immediately after immersion, the sensor was agitated for 4–5 s to facilitate hydration and dislodge the adsorbed water. OCP measurements began immediately following this step and were recorded over a 120-s interval. The response time was defined as the time required for the sensor to reach 90% of its new stable potential. After each measurement, the sensor was rinsed and transferred to the next solution, and the entire procedure was repeated. Each experimental condition was evaluated in triplicate to ensure reproducibility.

## Results and Discussion

SEM images reveal that the oxide layers on the WLa electrode (Figure 1a) and WTh electrode (Figure 1c) exhibit a seaweed-like morphology, which is consistent with findings reported by Mohamed et al.^15^ and Díaz-Ballote et al.^9^ Notably, the WLa sample has a greater density of cavities than the WTh sample, which appears more uniform and compact. This shows that the WLa surface is more porous, leading to a significant increase in surface area.

**Figure 1:**
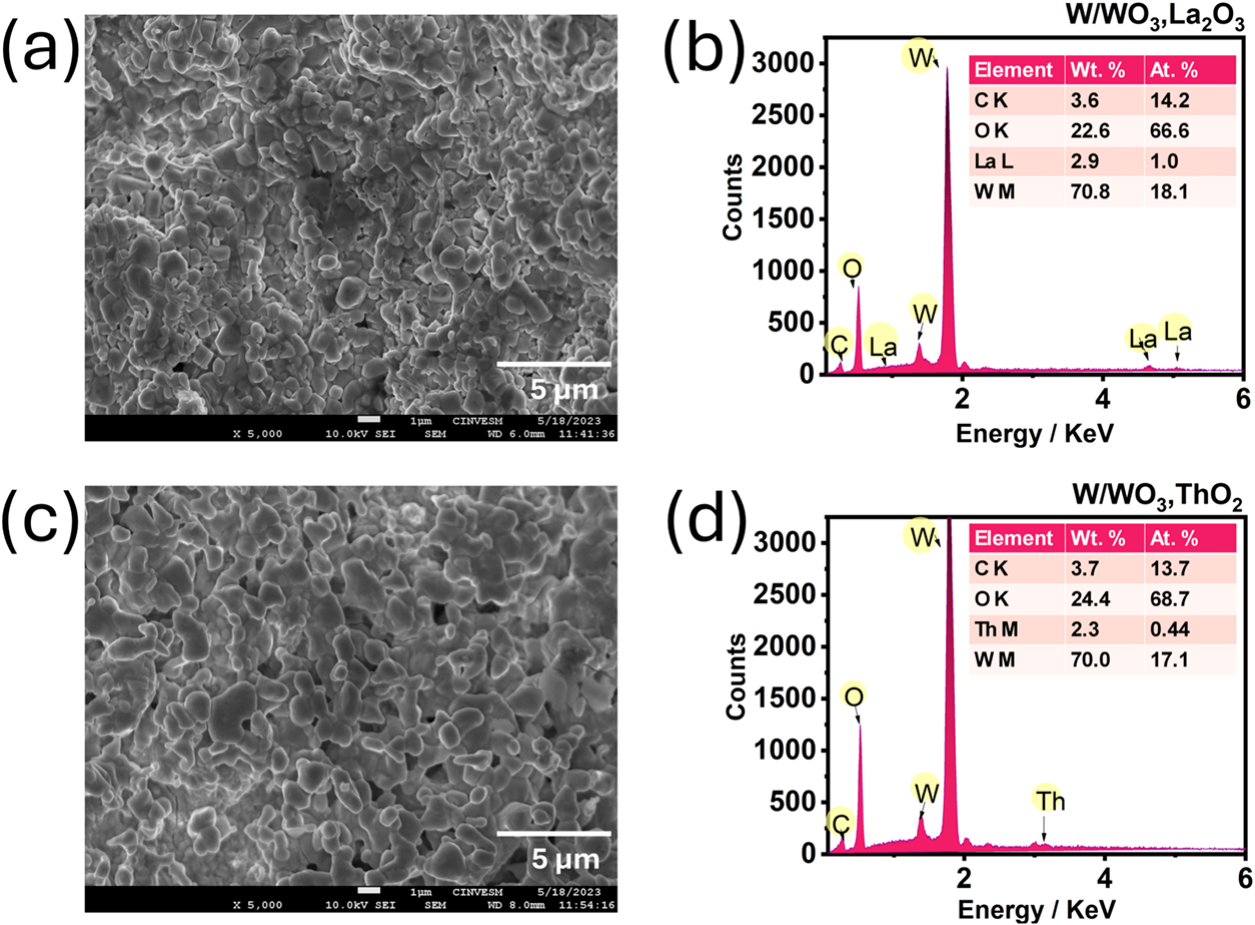
(a) SEM image showing the morphology of the WLa sample, with its corresponding (b) EDS plot and inset table displaying the quantitative element concentration. (c) SEM image showing the morphology of the WTh sample, with its corresponding (d) EDS plot and inset table displaying the quantitative element concentration.

The inset tables in Figure 1b,d present the elemental compositions of the WLa and WTh surfaces, respectively. The measured weight percentages of La and Th are 2.9% and 2.3%, respectively. Although these values do not exactly match the nominal 2% by weight of La_2_O_3_ and ThO_2_ reported for tungsten electrodes, the quite slight differences are reasonable given the initial dispersion of the oxides in the lanthanated and thoriated electrodes. The detected carbon is attributed to the use of carbon tape to fix the samples.

### XPS Analysis

Tungsten (W), atomic number 74, with the electronic configuration [Xe] 4f^14^ 5d^4^ 6s^2^, forms tungsten trioxide (WO_3_), which shares structural similarity with rhenium trioxide (ReO_3_). In both oxides, the metal cation occupies the center of an octahedron surrounded by six oxygen atoms located at the vertices. However, when the structure of WO_3_ is compared with that of ReO_3_ and the ABO_3_-type perovskite structure, from which both WO_3_ and ReO_3_ can be derived by removing the A cation, WO_3_ results in greater structural distortions.^16^ These distortions are attributed to the relative positions or tilting of the octahedra and the nonideal positioning of the tungsten cation within the octahedral structure,^16,17^ which does not occupy the central sites perfectly. Figure 2 shows the monoclinic WO_3_ structure with slight distortions.^18,19^

**Figure 2:**
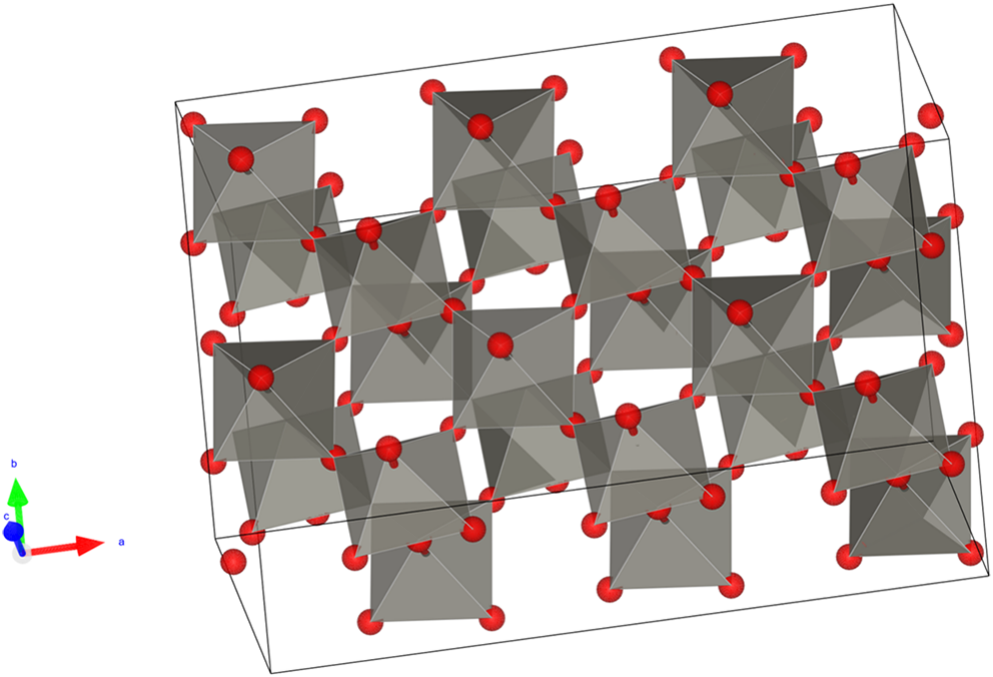
Monoclinic structure of tungsten trioxide.

In compounds such as tungsten trioxide (WO_3_), tungsten’s valence electrons are transferred to the oxygen atoms, leading to a final electronic configuration of 5d^0^, corresponding to the W^6+^ oxidation state.^20^ In X-ray photoelectron spectroscopy (XPS), this +6 oxidation state is characterized by a doublet corresponding to the 4f7/2 and 4f5/2 levels.^9^ Figure 3a displays the survey spectrum of the functionalized lanthanated tungsten electrode, confirming the presence of tungsten, lanthanum, and oxygen. Figure 3b shows the high-resolution spectrum, with peaks corresponding to the 4f state centered at binding energies of 37.6 and 35.4 eV, confirming the presence of WO_3_. The +6- oxidation state of tungsten in WO_3_ is the highest achievable;^20^ however, other oxidation states, such as W^5+^ and W^4+^ can also occur within the WO_3_ structure, particularly when oxides are exposed to elevated temperatures.^21^ These lower oxidation states arise from the partial reduction of tungsten, which is often associated with oxygen vacancies in the crystal lattice.^22,23^ In the XPS spectrum, a mixture of reduced tungsten cations such as W^5+^ and W^4+^ manifests as a peak in the lower energy region, such as the very weak peak centered at 33.4 eV shown in Figure 3b.^24–26^ Although this peak is barely distinguishable, it is observable, and its intensity is explained by the small amount of La_2_O_3_ (2%) originally dispersed in the tungsten electrode. The appearance of this weak peak suggests the presence of oxygen vacancies, which facilitate the reduction of W^6+^ to W^5+^ or even to W^4+^, indicating that the oxide film has mixed tungsten states, which also lead to the generation of free electrons.^22^ These free electrons enhance the electrical conductivity of the oxide, which could explain the improved sensor performance.

**Figure 3:**
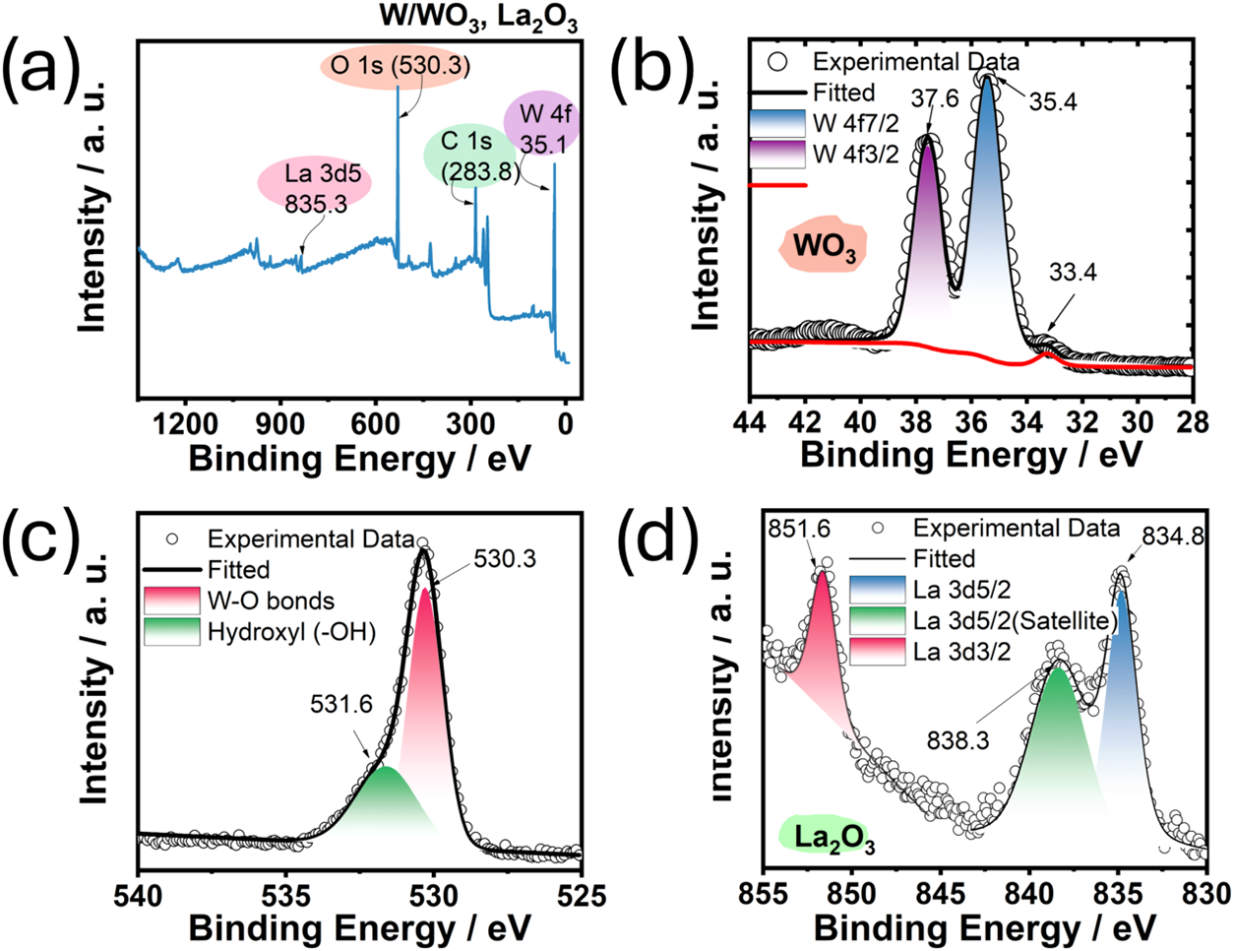
(a) XPS survey spectrum of the WLa surface. High-resolution spectra of (b) W 4f, (c) O 1s, and (d) La 3d of the WLa surface.

The asymmetry of the oxygen peak in Figure 3c suggests the need for deconvolution. The results revealed two peaks centered at 530.3 and 531.6 eV, corresponding to the W–O and W–OH bonds on the surface of the lanthanated electrode. The presence of lanthanum as lanthanum oxide (La_2_O_3_) is confirmed by peaks centered at 834.8, 838.3, and 851.6 eV (Figure 3d).

A similar analysis was performed for the thoriated tungsten electrode, with the results shown in Figure 4a–d. The survey spectrum reveals the presence of tungsten, oxygen, and thorium (Figure 4a). The oxidation state of each element was examined via XPS. Figure 4b confirms the presence of tungsten oxide based on the peaks centered at 35.4 and 37.5 eV. Figure 4c shows the oxygen peaks centered at 530.1 and 531.5 eV, corresponding to the W–O and W–OH bonds on the surface of the thoriated electrode. The presence of thorium oxide is indicated by peaks centered at 335.0 and 344.3 eV (Figure 4d). No significant peaks were observed in the low-energy region, suggesting fewer oxygen vacancies in the thoriated electrode.

**Figure 4:**
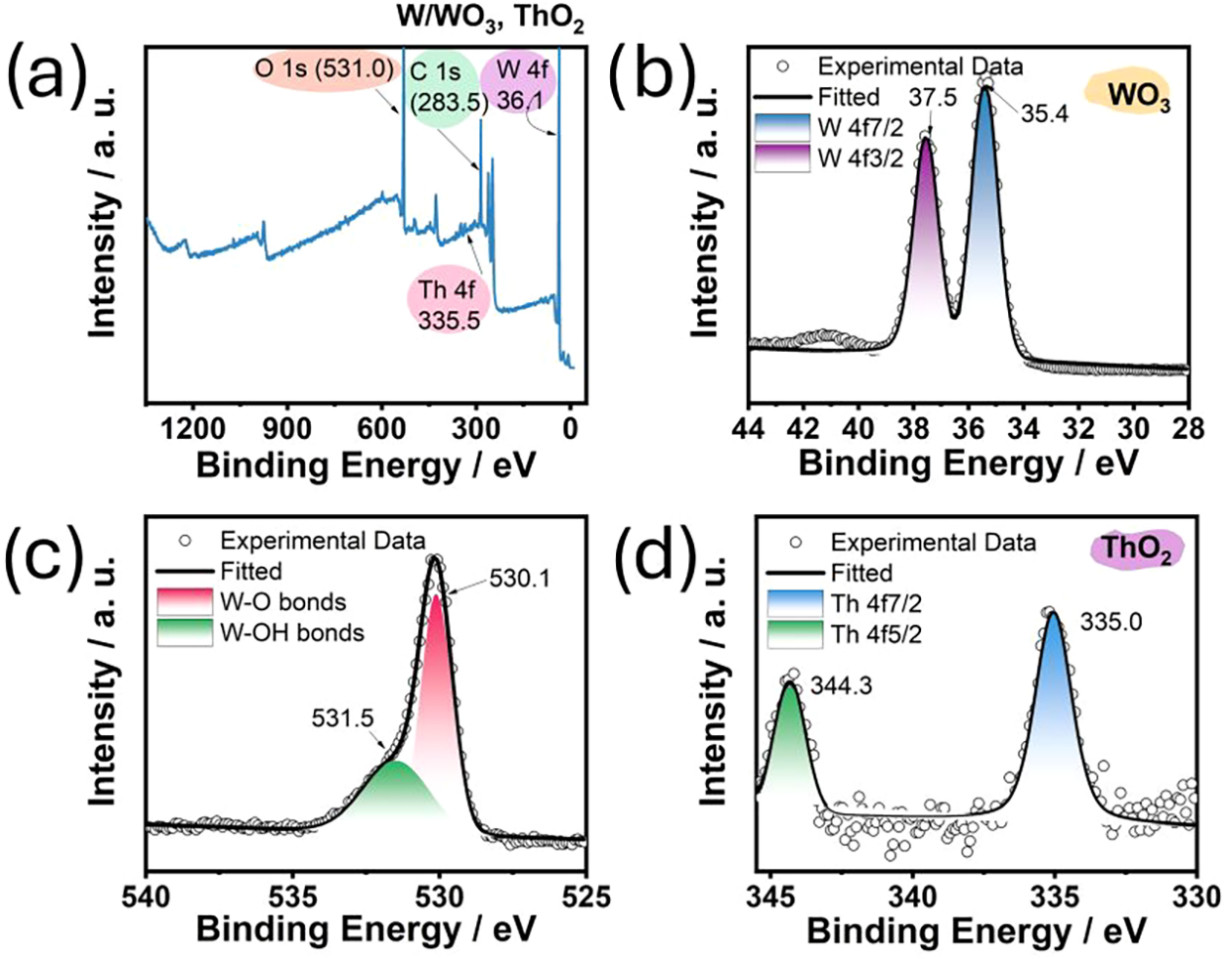
(a) XPS survey spectrum of the WTh surface. High-resolution spectra of (b) W 4f, (c) O 1s, and (d) Th 3d of the WTh surface.

### Lanthanated Electrodes as pH Sensors

Figure 5 shows the performance of the functionalized lanthanated tungsten electrode (WLa) as a pH sensor, highlighting its electrochemical behavior in response to the solution pH. In Figure 5a, the WLa electrode potential response is depicted with an Ag/AgCl electrode used as a reference. The observed potential shifts significantly as a function of pH, demonstrating the clear sensitivity of the electrode to changes in H^+^ concentration.

**Figure 5:**
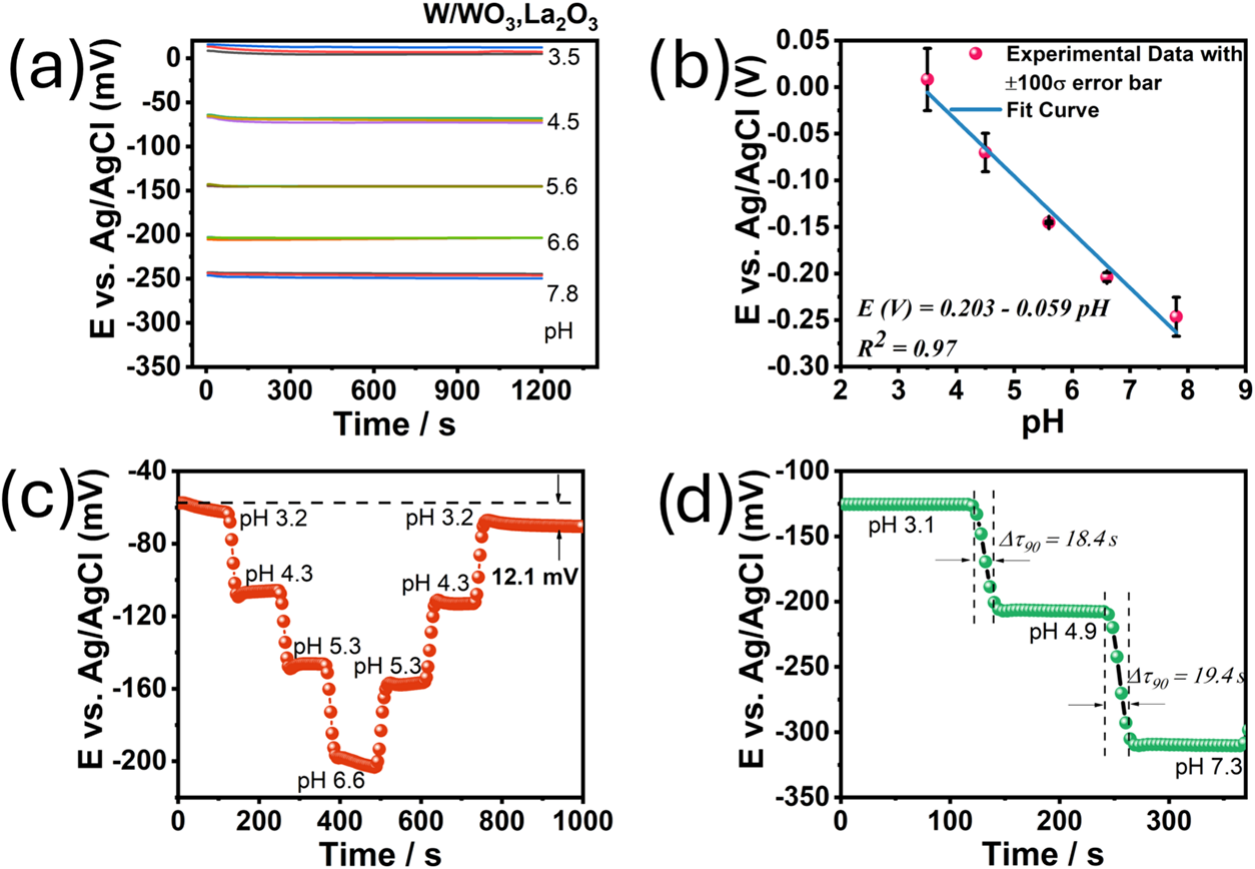
Electrochemical performance of the WLa lanthanated electrode. (a) Open-circuit potential as a function of time, (b) potential vs pH dependence, (c) hysteresis width of WLa at pH 3.2–4.3–5.3–6.6 and 6.6–5.3–4.3–3.2 loop cycles, (d) results from response time experiments.

As shown in Figure 5b, a strong linear correlation (*R* ^2^ = 0.97) between the electrode potential and pH was established, with a measured sensitivity of −58.8 mV at pH^–1^. This value is slightly below the theoretical Nernstian sensitivity of −59.0 mV pH^–1^, indicating excellent performance. Notably, this sensitivity is better than the sensitivity of −40.0 mV pH^–1^ previously reported for pure WO_3_,^9^ highlighting the significant improvement in the materials and functionalization methods used.

The hysteresis of the WLa electrode was also assessed, as depicted in Figure 5c. In this experiment, the pH was adjusted to various values by adding precise amounts of stock solution, and the corresponding electrode potential was recorded. The system was returned to the initial pH value following the same procedure to measure hysteresis. The hysteresis was approximately 12.1 mV, which aligns with previously reported values for functionalized metal oxide electrodes,^27,28^ including WO_3_ (13 mV).^9^ This low hysteresis value suggests good reproducibility and stability of the sensor, which is critical for applications requiring precise pH measurements.

The response time, another key parameter for evaluating sensor performance, is estimated in Figure 5d. In this experiment, three solutions with pH values of approximately 3, 5, and 7 were used. Prior to immersion in each solution, the electrodes were rinsed with distilled water. Once submerged, the solution was gently stirred for 5 s to remove residual distilled water from the electrode surface, after which the electrode potential was recorded. The response time was defined as the duration required to reach 90% of the steady state potential. The electrode showed response times ranging from 17.3 to 19.8 s, which is well suited for pH sensors, particularly for those used in real-time monitoring applications.^29^ The response time observed for WLa in the present work falls within the same range as that of WO_3_, which was previously reported to be between 18.4 and 19.4 s.^9^ This range of response times is advantageous in dynamic environments where rapid pH fluctuations occur.

In general, the WLa electrode offers several advantages, including ease of functionalization, reasonable sensitivity, low hysteresis, and quick response time. Furthermore, its low-cost fabrication and adaptability make it a competitive option for practical pH sensing applications.

### Thoriated Electrode as a pH Sensor

Figure 6 shows the performance of the thoriated tungsten electrode (WTh) as a pH sensor, highlighting its electrochemical response to various pH values. In Figure 6a, the potential response of the WTh electrode vs the Ag/AgCl reference electrode is shown. Like the WLa electrode, the WTh electrode is sensitive to changes in H^+^ concentration, as demonstrated by the shift in potential in response to varying pH levels.

**Figure 6:**
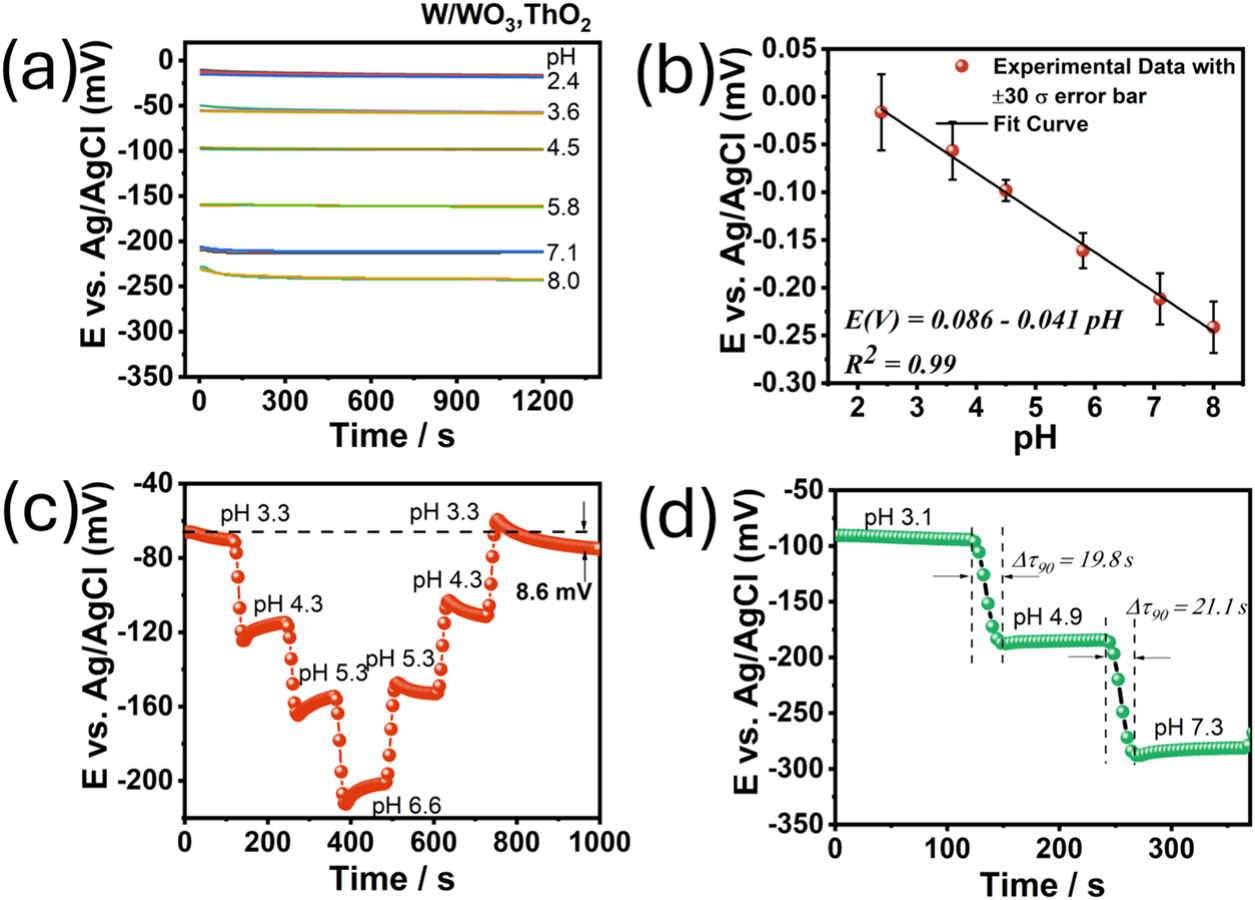
Electrochemical performance of the thoriated WTh electrode. (a) Open-circuit potential as a function of time, (b) potential vs pH dependence, (c) hysteresis width of WTh at pH 3.2–4.3–5.3–6.6 and 6.6–5.3–4.3–3.2 loop cycles, (d) results from response time experiments.

Figure 6b shows the relationship (*R* ^2^ = 0.98) between the electrode potential and pH, with a measured sensitivity of −41.6 mV at pH^–1^. Although this value is lower than that observed for the WLa electrode (−58.8 mV pH^–1^), it still demonstrates reasonable sensitivity for pH sensing applications. The sub-Nernstian sensitivity of the WTh electrode is often attributed to interference from dissolved ions and oxygen in the test solution, as well as factors such as surface interactions with analytes.^9,30^ Despite its reduced sensitivity compared with the WLa electrode, the WTh electrode remains functional and may still be suitable for applications requiring high-precision pH measurements.

Hysteresis, another important parameter, is depicted in Figure 6c. The WTh electrode has a hysteresis value of 8.6 mV, which is notably lower than the 12.1 mV observed for the WLa electrode. This reduced hysteresis implies better reproducibility and stability in the WTh electrode, which could make it advantageous in applications where minimal drift is critical.^31^ However, it is important to note that the lower hysteresis comes at the cost of slightly reduced sensitivity.

Figure 6d shows the response time of the WTh electrode, which ranged between 19.8 and 21.1 s. This is comparable to the response time of the WLa electrode (18.4 to 19.4 s). Both electrodes show adequate response times for real-time pH monitoring.

When the overall performance of the WTh electrode is compared with that of the WLa electrode, the WLa electrode exhibits superior sensitivity (−58.8 versus −41.6 mV pH^–1^), making it more appropriate for applications requiring high precision. On the other hand, the WTh electrode’s lower hysteresis value (8.6 mV) suggests that it may offer more stable and reproducible measurements over time, albeit with a trade-off in sensitivity. Thus, while both electrodes are viable for pH sensing applications, the WLa electrode may be preferred in cases where high sensitivity is crucial, whereas the WTh electrode might be more suitable in environments where stability and reproducibility are prioritized.

To place these findings in a broader context, it is useful to consider the performance of conventional pH sensors. Commercial glass electrodes are still the reference for pH sensing, providing near-Nernstian slopes (∼59 mV pH^–1^ at 25 °C) and long-term stability.^32^ However, their use can be limited by their mechanical fragility, relatively large dimensions, and difficulties in miniaturization or integration under harsh conditions. In contrast, WO_3_-based electrodes reported in the literature^1^ display comparable Nernst-like sensitivities and rapid responses (often <10 s in optimized thin films), whereas hysteresis and drift vary depending on the fabrication method (e.g., ∼15.7 mV h^–1^ for RF-sputtered films versus <3 mV for magnetron-sputtered films).^1^ Within this context, our functionalized electrodes exhibited sensitivities of −58.8 mV pH^–1^ (WLa) and −41.6 mV pH^–1^ (WTh), with response times of ∼18–19 s and ∼20–21 s and hysteresis values of ∼ 12 mV and ∼ 9 mV, respectively. These values fall within the range reported for WO_3_ electrodes, highlighting their potential as robust alternatives in applications where durability and miniaturization are important. All buffer solutions were also verified via a commercial pH electrode (HI98103, Hanna Instruments), ensuring consistency with conventional pH measurement practices.

### Mechanisms for pH Sensitivity in WO_3_

The pH sensitivity of tungsten trioxide (WO_3_) results from a combination of surface and bulk processes, whose relative contributions depend on several factors, such as the crystal phase, morphology, dopant chemistry, and synthesis conditions. Several mechanisms have been proposed to explain this sensitivity. None alone fully explain all observations reported in the literature, suggesting that multiple pathways may operate simultaneously or under different experimental conditions.

One widely reported non faradaic mechanism is surface hydroxyl ion exchange. In this process, – OH groups form on the WO_3_ surface through the dissociative adsorption of water.^33–36^ These hydroxyl groups can undergo protonation or deprotonation (eqs 1 and 2):

1 $$\mathrm{WOH}+H^{+}⇌{\mathrm{WO}H_{2}}^{+}$$

2 $$\mathrm{WOH}⇌{\mathrm{WO}}^{-}+H^{+}$$

The equilibrium between these protonated and deprotonated sites modifies the surface charge and consequently the interfacial potential, producing a measurable pH-dependent signal. The magnitude of this response is determined by the density of active sites and the dielectric properties of the tungsten oxide, both of which are strongly influenced by synthesis and posttreatment. This mechanism often yields slopes near Nernstian slopes (∼59 mV pH^–1^ at 25 °C).^33^

A dominant faradaic pathway in WO_3_ is the well-known proton intercalation mechanism. In this process, protons from the electrolyte and electrons from the electrode are intercalated into the WO_3_ lattice to form tungsten bronzes (H_*x*_WO_3_) a reaction described by eq 3:^5,37–39^

3 $${\mathrm{WO}}_{3}+x\;H^{+}+x\;e^{-}⇌H_{x}{\mathrm{WO}}_{3}$$

This proton intercalation changes the oxidation state of tungsten (W^6+^ → W^5+^, or even W^4+^) and alters the electronic structure, often shifting the Fermi level toward the conduction band and therefore increasing the electrical conductivity.^40^ Structural relaxation frequently accompanies the process, and in some cases, a monoclinic-to-cubic phase change occurs, enhancing proton mobility and improving sensing performance. Because proton intercalation involves coupled electron transfer, it is thermodynamically equivalent to the so-called proton-coupled redox equilibrium described in the literature.^41^

The oxygen–vacancy formulation is an alternative way of expressing the same coupled proton–electron process, focusing on the oxygen balance within the oxide lattice:^42^

4 $${\mathrm{WO}}_{3}+2\delta \;H^{+}+2\delta \;e^{-}⇌{\mathrm{WO}}_{3-\delta }+\delta \;H_{2}O$$

Equation 4 shows that protons and electrons remove lattice oxygen as water does, creating oxygen vacancies (δ). While under mild sensing conditions, δ may be negligible, under more reducing conditions or prolonged cycling, vacancy formation can become significant and influence the pH response of the electrode.

A less desirable but possible contributor is the steady-state corrosion mechanism.^43^ In alkaline media, WO_3_ can dissolve to form soluble tungstate species according to the eq 5:^44–46^

5 $${\mathrm{WO}}_{3}\left(s\right)+H_{2}O⇌{\mathrm{WO}}_{4}^{2-}\;\left(\mathrm{aq}\right)+2\;H^{+}$$

In this case, the electrode potential reflects a mixed potential derived from the balance between pH-dependent anodic dissolution and a cathodic process such as the reduction of oxygen. While this pathway can influence long-term stability, it is generally detrimental for sensing applications because material loss because the corrosion process is effectively irreversible under operating conditions.

From a computational perspective, density functional theory (DFT) has been useful in clarifying aspects of the proton intercalation mechanism. Simulations show that proton insertion is always accompanied by electron injection, reducing the tungsten oxidation state and shifting the Fermi level to the conduction band.^40^

Despite extensive phenomenological and computational studies, these mechanisms remain partially validated, and contradictions persist in theoretical descriptions for doped tungsten, such as WLa and WTh, for which no comprehensive theoretical framework exists. This is mainly because computational predictions are sensitive to assumptions about structure and morphology, making direct comparison with experimental data challenging. Considering the mechanisms described in the literature and our XPS analysis, the mechanism with the highest probability of explaining our observations is proton intercalation into the WO_3_ lattice. Nevertheless, this hypothesis should be interpreted as the most plausible among competing models, rather than as a definitive explanation, given the current state of theoretical knowledge.

### pH Measurement of Real Samples

To evaluate the sensor performance of the WLa and WTh electrodes, pH measurements were conducted on various real-world samples, including apple juice, vinegar, drinking water, and light milk, each with distinct pH values. Before each measurement, the electrodes were rinsed thoroughly with deionized water to avoid cross-contamination.

For validation, a commercial pH meter (HI98103, Hanna Instruments; resolution: 0.1 pH, accuracy: ± 0.2 pH) was used concurrently to measure the pH of the same samples. The WLa and WTh electrodes were immersed in the samples for 3 min, after which the recorded pH values were compared with those obtained from a commercial pH meter. The electrodes were calibrated prior to use, with sensitivities of −58.8 mV pH^–1^ for the WLa electrode and −41.6 mV pH^–1^ for the WTh electrode.

The results, summarized in Table 1, indicate good agreement between the pH values measured by the electrodes and those recorded via the commercial pH meter. This consistency extends even to near-neutral pH samples, such as light milk. Measuring the pH of milk typically poses challenges because of the need for longer stabilization times, as milk components—particularly proteins and fats—can adsorb onto the electrode surface and hinder ion diffusion.^47^ Despite these challenges, WLa and WTh electrodes have demonstrated reliable performance.

**Table 1:** pH of Various Real-World Samples^a^

sample	pH meter	WLa pH (%RSD)	WTh pH (%RSD)
apple juice	4.2	4.5 (0.5)	4.3 (0.1)
vinegar	3.3	3.6 (1.8)	3.5 (0.9)
light milk	6.6	6.8 (1.0)	6.3 (0.7)
drinking water	6.2	6.5 (0.2)	6.2 (0.6)

a Percent relative standard deviation, %RSD (*n* = 3 measurements).

The maximum difference observed between the commercial pH meter and the oxide-based sensors was less than 0.3 pH units, highlighting the potential of these electrodes for practical pH measurement in real-world samples. Furthermore, the relative standard deviation (RSD) of less than 3% across three measurements (n = 3) for all investigated samples confirms the stability of the electrode potential over time.

## Conclusions

This study demonstrated that lanthanated (WLa) and thoriated (WTh) tungsten electrodes are promising alternatives to conventional glass pH sensors. The WLa electrode exhibited high sensitivity (−58.8 mV pH^–1^), low hysteresis (12.1 mV), and fast response (18–20 s), which was attributed to its porous structure and the presence of oxygen vacancies that enhance proton interaction and conductivity. The WTh electrode, while slightly less sensitive (−41.6 mV pH^–1^), showed minimal hysteresis (8.6 mV), making it suitable for applications where this parameter is critical.

Despite these encouraging results, the current findings are limited to controlled laboratory conditions. Further studies are needed to evaluate long-term stability, sensor behavior in aqueous media containing interfering ions, and response time under variable temperature.
